# Comparison efficacy and safety of acupuncture and moxibustion therapies in breast cancer-related lymphedema: A systematic review and network meta-analysis

**DOI:** 10.1371/journal.pone.0303513

**Published:** 2024-05-14

**Authors:** Yawen Xu, Jiangxuan Yu, Rui Shen, Xueqi Shan, Wenlu Zhou, Junjie Wang

**Affiliations:** School of Nursing, Zhejiang Chinese Medical University, Hangzhou, Zhejiang, China; Chinese University of Hong Kong, HONG KONG

## Abstract

**Objective:**

Although several acupuncture and moxibustion therapies have been tested in managing breast cancer-related lymphedema (BCRL), there is little consensus regarding the best options for treating this condition. This systematic review and network meta-analysis compared the efficacy of various acupuncture and/or moxibustion therapies for BCRL.

**Methods:**

Seven databases and two clinical registration centers were searched from their inception to December 1^st^, 2023. The Cochrane Collaboration risk-of-bias assessment tool evaluated the quality of included RCTs. A pairwise meta-analysis was performed in STATA 16.0, while a network meta-analysis was performed in R 4.2.2.

**Results:**

18 studies were included in this analysis. Our results showed that acupuncture and moxibustion methods had great advantages in improving BCRL of patients with breast cancer. In particular, needle-warming moxibustion (NWM) could be the optimal acupuncture and moxibustion method for improving clinical effectiveness and reducing the degree of swelling of affected limbs.

**Conclusion:**

Our findings suggest that NWM has great potential in treating BCRL. It may reduce arm circumference, lower swelling levels, and improve clinical effectiveness. Nevertheless, more multi-center, high-quality, and large sample RCTs will be needed in the future.

## Introduction

Breast cancer (BC) is a malignancy with the highest incidence in women worldwide. Benefiting from medical advances, postoperative survival is prolonged, and the patient’s quality of life is getting more attention. Breast cancer-related lymphedema (BCRL), usually a temporary or sustainable soft tissue swelling caused by the excessive accumulation of protein-rich lymph in the extracellular spaces of tissue [1], which manifests as numbness, swelling, pain, dysfunction, and even infection, is a common complication after surgery, radiotherapy, or chemotherapy. The incidence of BCRL in BC survivors ranges from 7% to 58% because of different treatments [2, 3]. Although the pathogenesis of BCRL is still incompletely understood, it involves a series of continuous and complex processes including fat deposition, lymphatic fibrosis, and infiltration of inflammatory cells [4]. As a serious complication, BCRL has not been well recognized and has not been effectively resolved.

Surgical and non-surgical therapies are now available for BCRL. Non-surgical therapies mainly include physical therapy and drugs, but drugs (Diosmin or diuretics) may be limited in clinical practice due to slow reaction times, and adverse effects. Combined decongestive therapy (CDT) is recognized as the recommended physical therapy for lymphedema, which consists of manual lymphatic drainage (MLD), gradient compression bandaging (GCB), functional exercises, and skin care [5]. However, CDT is an expensive and time-consuming strong treatment method that requires long-term daily one-on-one intervention with a professional therapist, so it brings patients inconvenience and discomfort [6]. Therefore, considering the current clinical conditions and patient adaptability, for patients with mild to moderate edema, some relatively simple methods such as functional exercise (FE) and pneumatic circulation (PC) are adopted. Whereas for severe cases or those with ineffective CDT, surgery, like lymphatic venous anastomosis (LVA) or vascularized lymph node transfer (VLNT), is the only and final option [7]. Nevertheless, the results of these methods are very limited and slow, and surgeries may lead to recurrence or infection [8]. Therefore, it is crucial that there are alternate therapies available.

As complementary or alternative therapies for BCRL, acupuncture and/or moxibustion therapies, using metal fine needles or moxa sticks to stimulate acupoints for therapeutic purposes, have been recommended to relieve the symptoms of BCRL [9]. According to several meta-analyses, acupuncture and moxibustion therapies show strong potential advantages for the management of BCRL [10, 11]. According to a study, local acupuncture might accelerate metabolism, increase blood circulation, and cause accumulation of inflammatory cells in the upper limb edema areas to attempt to mitigate the edema [12]. Acupuncture’s stimulatory impact leads the damaged or blocked lymphatic vessels to reopen, particularly the micro-vessels that are essential for lymphatic return [13]. Moxibustion’s warm stimulation could effectively alleviate the pain and promote the healing process [14].

However, there are various acupuncture and moxibustion techniques, each with varying levels of complexity, costs, as well as safety and efficacy [15, 16]. Appropriate acupuncture and moxibustion modality selection can enhance therapeutic outcomes and lower treatment costs, which in turn lowers the strain on clinicians and patients. However, due to insufficient evidence of direct comparisons between acupuncture and moxibustion therapies, clinicians find it difficult to determine which are the most effective for clinical applications [17]. Network meta-analysis (NMA), in comparison to conventional meta-analysis, may compare different treatments for one health condition and perform quantitative analysis to evaluate and order the efficacy of these therapies [18]. Therefore, a systematic review and network meta-analysis were used to compare and rank different acupuncture and moxibustion methods, to identify the most effective acupuncture and moxibustion methods and provide a reference for clinical practice.

## Methods

Our network meta-analysis has been registered on the PROSPERO website (CRD42023392176) and reported following the Preferred Reporting Items for Systematic Reviews and Network Meta-Analysis (PRISMA-NMA) checklist [19] (S1 Table).

### Search strategy

We searched PubMed (with MEDLINE), Web of Science, Embase, China National Knowledge Infrastructure (CNKI), Wanfang, China Science and Technology Journal Database (VIP), and SionMed databases to identify relevant English/Chinese medical studies. The search strategy combined MeSH terms with free-text terms. The keywords include “breast cancer”, “breast cancer-related lymphedema”, “lymphedema”, “acupuncture”, and “moxibustion”, as well as their relevant derivatives. Boolean “OR” and “AND” were used to find the intersections of searches for breast cancer with acupuncture and moxibustion. The search period ran from the database’s establishment until December 1^st^, 2023. Comprehensive search strategies were provided in the S2 Table.

Additionally, we searched published meta-analyses, ClinicalTrials, Chinese Clinical Trial Registry (ChiCTR), and gray literature. Two researchers examined the findings for accuracy and looked through the included trials’ reference lists.

### Inclusion and exclusion criteria

#### Inclusion criteria

Studies were deemed qualified if they met all of the specified eligibility criteria (PICOs) described below:

1. Type of participants: Women who had lymphedema as a result of surgery, chemotherapy, or radiation for BC. There were no limitations on participants’ lymphedema level, BC stage, age, or nation.
2. Type of interventions: The treatment group was given acupuncture and/or moxibustion without the restriction of manipulation techniques, but in the moxibustion measures, only moxa was heated without Traditional Chinese medicines.
3. Type of comparators: The control group could receive acupuncture and/or moxibustion (different from the intervention group) or any other treatments (such as oral medication, physical therapy, placebo acupuncture, and so on).
4. Type of outcomes:

The primary outcomes were clinical effectiveness rate and extent of lymphedema evaluation using the arm circumference (average arm circumference, circumference of elbow joint). The clinical effectiveness rate was determined based on the effective index (%): Effective index (%) = (pretreatment circumference of the affected arm—posttreatment circumference of the affected arm) / (pretreatment circumference of the affected arm—pretreatment circumference of the unaffected arm). There are two methods for calculating:

- Significantly effective: effective index > 90% or above; effective: 10–90%; ineffective <10%. Clinical effectiveness rate = significantly effective + effective.
- Cure: reduction of 75% or above; significantly effective: reduction of 51–74%; effective: reduction of 25–50%; ineffective: reduction of less than 25%. Clinical effectiveness rate = cure + significantly effective + effective.

The secondary outcomes were composed of the visual analog scale (VAS) score for swelling or pain, and the incidences of adverse events related to acupuncture and moxibustion therapies (such as local skin discomfort, mild scald, vertigo headache, flushing, and itching headaches).

(5) Type of study: We only considered randomized controlled trials (RCTs).

#### Exclusion criteria

The study was excluded if it matched any of the criteria below: (1) duplicate publication, (2) no relevant outcomes, (3) unobtainable original full text, and (4) pretrial (pilot study), animal experiments, letters, reviews, commentaries, protocols, or conference presentations.

### Study selection and data extraction

Two researchers independently screened the literature, extracted the data, and conducted cross-checking. If there were any differences, a third researcher was invited to participate in the discussion to conclude. For the included studies, Excel 2021 software was used to extract the following information: basic study features (first author’s name, publication year), information regarding the participants, treatments, and outcomes. If the data were lacking or unclear, the author would be contacted to obtain the necessary information.

### Quality assessment

We evaluated the included RCTs’ bias risk through the Cochrane Collaboration risk-of-bias assessment tool [20] by using the Review Manager (version 5.3) with six assessment aspects. The classifications of "low risk," "unclear risk," and "high risk" were displayed for each study in green, yellow, and red, respectively. If the two reviewers’ assessments disagreed, the entire team voted and discussed in order to reach a conclusion.

### Statistical analysis

Firstly, a pairwise meta-analysis of direct comparisons was carried out on trials with clinical homogeneity due to the use of the same type of therapies, participants, and indicator evaluation methods using Stata 16.0 software. For dichotomous outcomes, the effect size was represented using odds ratios (ORs) and 95% confidence intervals (CIs). Statistical significance was defined as P < 0.05. Weighted mean differences (WMDs) and 95% CIs were estimated for continuous outcomes. To examine the heterogeneity of included studies, we used the I^2^ test. If the heterogeneity was non-significant (P value ≧ 0.1 and I^2^ ≦ 50%), we used a fixed effect model. Conversely, a random-effect model was adopted, and then the sources of heterogeneity were explored [21].

Secondly, using the “BUGSnet” and “gemtc” packages in R 4.2.2 software, network meta-analysis was carried out and assessed with Markov Chain Monte Carlo (MCMC) simulation [22, 23]. To evaluate model fit, we compared the number of unconstrained data points and the residual deviation. If the quantities were nearly the same, the model fit would be regarded as sufficient. The deviation information criterion (DIC) served as the basis for our decision on whether to use a fixed-effect or a random-effect model. We selected a model with lower DIC values. However, if the DIC values of the two models were close and their difference was within 5, a random-effect model could be chosen. Gelman and Rubin criteria as well as a review of trace plots would be used to assess convergence. The network plot of the outcome was made to visualize multiple comparisons. Consistency was evaluated by node splitting [24], and inconsistency was defined as P value < 0.05. A heatmap with all potential comparisons was used to display the relative effect estimate from this analysis. For treatment ranking, the surface under the cumulative ranking curve (SUCRA), which ranges from 0% to 100%, was calculated. When comparing the various therapies, the intervention with the highest SUCRA value has the highest possibility that it would be the best one [25].

In addition, if there were more than 10 studies included in each outcome, comparison-adjusted funnel plots and Egger’s tests were conducted to assess publication bias.

## Results

### Study selection and study characteristics

**Fig 1:**
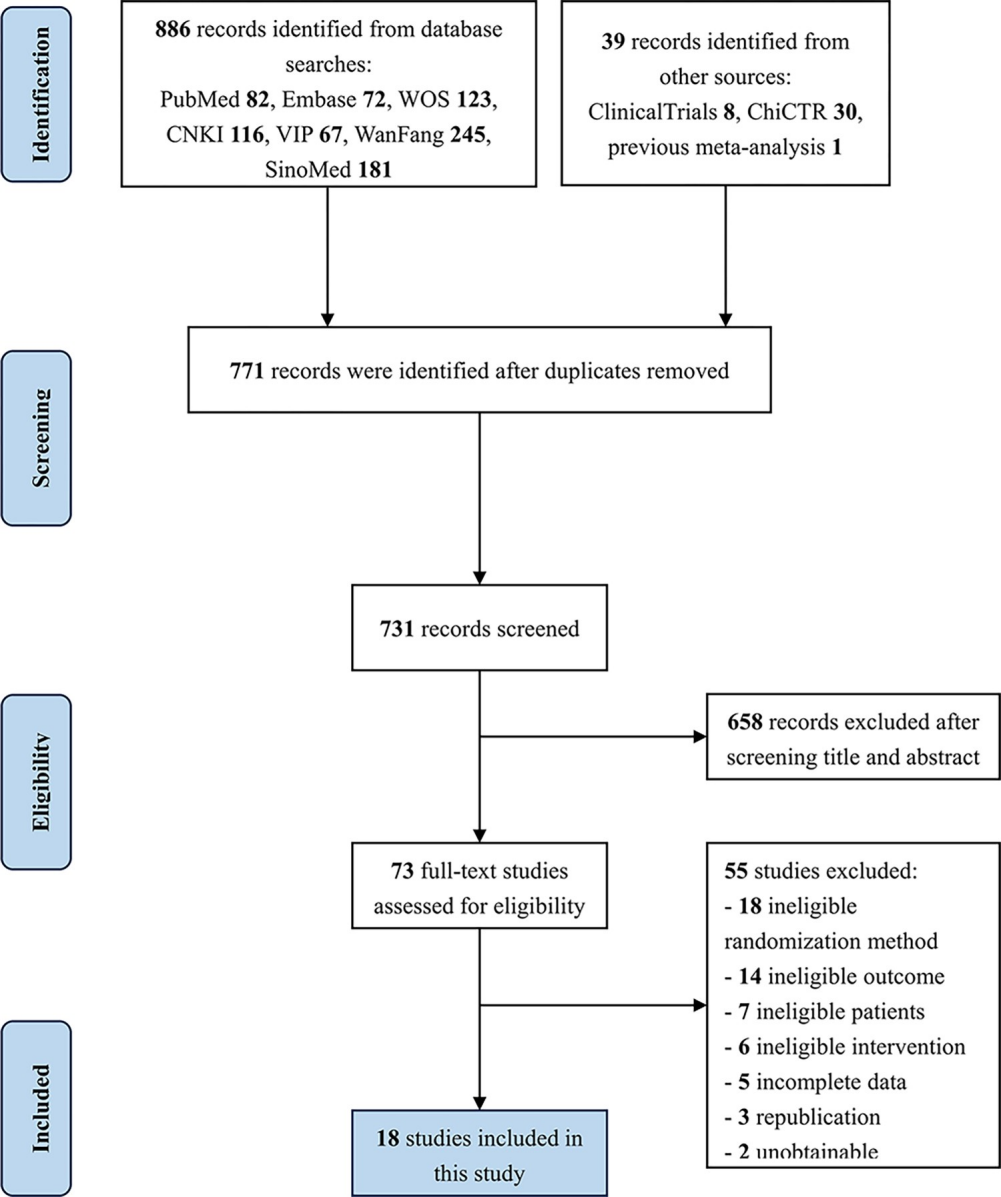
PRISMA flow chart.

The screening process is presented in Fig 1. In the end, we identified 886 related studies during our initial evaluation. After duplicates were eliminated and titles and abstracts were screened, 73 trials that may meet the criteria were read carefully in full text. Finally, our quantitative synthesis included 18 RCTs in total.

The studies, involving 1,217 patients and published from 2014 to 2023, included 5 acupuncture and/or moxibustion treatments, namely simple acupuncture (SA), blood-letting and cupping (BLC), electroacupuncture (EA), gentle moxibustion (GM) and needle-warming moxibustion (NWM), as well as 5 comparators consisting of FE, oral medication (OM), PC, placebo acupuncture (PA), and usual care (UC). Table 1 provides further information. The complete relevant data is shown in S1 File.

**Table 1:** Characteristics of the included studies.

Study ID	N	Age	Diagnosis	Treatment	Primary acupoints	Retention(min)	Course	Outcomes
Hu [26]	39	46.00±7.50	Ⅰ	GM	LI.11, LI.15, SP.10, SP.9, ST.36	25	4 weeks, Qd	①
	39	45.60±8.90		FE		20	4 weeks, Bid	
Zhang [27]	14	57.25±7.19	Ⅰ	GM	LI.14, LI.11, CV.3, SI.9, and Ashi points.	30	4 weeks, Biw	①②④
	14	58.36±8.12		PC		-	4 weeks, Biw	
Liu [28]	30	55.00±11.00	Ⅰ	NWM	LI.4, LI.11, LI.15, GB.21, SP.9, CV.9, TE.5, TE.14, and Ashi points.	30	6 weeks, Qd	①④
	30	55.00±10.00		UC		-	6 weeks	
Ba [29]	30	47.40±4.30	Ⅰ	NWM	GB.34, HT.1, LI.4, LI.10, LI.11, LI.14, LR.3, TE.5, ST.36, SP.6, SP.9.	20–30	14 days, Qd	②③⑥
	28	46.30±3.20		OM		-	14 days, Bid	
Shen [30]	22	59.67±5.38	Ⅰ	GM	CV.4, LI.14, TE.5, TE.14.	20	2 months, 6 times per month	②③④⑥
	21	56.88±5.97		PC		30	2 months	
Wang [31]	24	59.42±7.02	Ⅰ	GM	BL.23, LI.13, LI.14, TE.5, SI.9, and Ashi points.	30	4 weeks, Q2d	②③④⑥
	24	58.25±6.19		PC		-	4 weeks, Q2d	
Xing [32]	20	61.21±4.21	Ⅲ	SA	LI.4, LI.11, LI.14, LI.15, LU.7, SI.9, SP.6, ST.36, TE.14, TE.6.	30	10 days, Qd	①
	20			UC		-	10 days	
Huang [33]	33	49.58±6.61	Ⅲ	EA	6–8 tendon attachment points of affected upper limb.	30	4 weeks, 6 times per week	⑥
	30	50.37±6.52		FE		-	4 weeks Qd	
Feng [34]	46	51.66±7.24	Ⅲ	BLC	BL.28, CV.3, CV.6, LI.4, LR.2, LR.3, ST.36, SP.6, SP.10, BL.17, SI.6.	20	28 days, Qd	③⑥
	47	52.17±6.03		OM		-	28 days, Bid	
Zhan [35]	30	57.84±4.40	Ⅰ	SA	CV.12, CV.10, CV.6, CV.4, ST.24, ST.26.	-	28 days, Qd	①⑤
	30			FE		-	4 weeks, Tiw	
Yang [36]	23	58.25±6.19	Ⅰ	GM	LI.14, LI.11, CV.3, SI.9, and Ashi points.	30	4 weeks, Biw	②③④⑥
	22	59.42±7.02		PC		-	4 weeks, Biw	
Sun [37]	10	44.20±8.60	Ⅲ	BLC	Three Yin and Three Yang Meridians; Du Meridian.	30	9 weeks, Tiw	①③⑤
	10	46.40±9.10		UC		-	9 weeks	
Jiao [38]	15	45.62±3.52	Ⅲ	NWM	GB.21, LI.4, LU1, LR.3, SP.9, TE.5, ST.36, and Ashi points.	20	9 weeks, 5 times per week	③
	15			UC		-	9 weeks	
Zhang [39]	48	59.90±7.02	Ⅰ	BLC	Three hand-yin and three hand-yang meridians, the Du meridian, the bladder meridian of foot-Taiyang.	10	50 days, every 5 days	①②③ ⑤⑥
	24	59.96±5.33		FE		30	50 days, Qd	
Huang [40]	31	50.00±10.00	Ⅲ	GM	CV.9, GB.21, LI.4, LI.11, LI.14, LI.15, LU.7, TE.5, TE.14, SP.9, SI.9, and Ashi points.	20	6 weeks, 5 times per week	①③
	31	50.00±9.00		UC		-	6 weeks	
Zhao [41]	36	53.00±9.00	Ⅰ	SA	LI.15, TE.5, LI.4, SP.9, ST.36, and Ashi points.	30	8 weeks, Tiw	①
	37	53.00±11.00		FE		10–15	8 weeks, Bid	
Yang [42]	48	61.31±9.64	Ⅰ	NWM	LI.4, LI.11, TE.5, TE.3, TE.9, LI.15, HT.13, TE.10, PC.2, CV.12, CV.4, LI.11, CV.9, SP.6, SP.9.	30	7 weeks, Tiw	④
	43	59.73±9.76		PA	1cm beside the upper and lower limb acupoints, and 2cm beside the abdominal acupoint.	30	7 weeks, Tiw	
Qiu [43]	30	58.10±9.87	Ⅲ	GM	BL.13, BL.22, SP.9, CV.6, LI.6, LI.4, SI.6, PC.6.	20–50	3 weeks, 5 times per week	③
	30	62.30±9.44		UC		-	3 weeks	

Ⅰ the injured limb’s circumference is more than 2 cm larger than the same area on the uninjured side; Ⅱ the injured limb’s circumference is more than 3 cm larger than the same area on the uninjured side; Ⅲ diagnosis method not specified. SA = simple acupuncture; BLC = blood-letting and cupping; EA = electroacupuncture; GM = gentle moxibustion; NWM = needle-warming moxibustion; FE = functional exercises; PC = pneumatic injection; OM = oral medicine; PA = placebo acupuncture; UC = usual care (comprehensive treatments including life care, health education, skin care, and so on). ① clinical effectiveness rate; ② average arm circumference; ③ circumference of the elbow joint; ④ VAS swelling score; ⑤ VAS pain score; ⑥ adverse events. The full names of acupoints abbreviations are shown in S3 Table.

**Fig 2:**
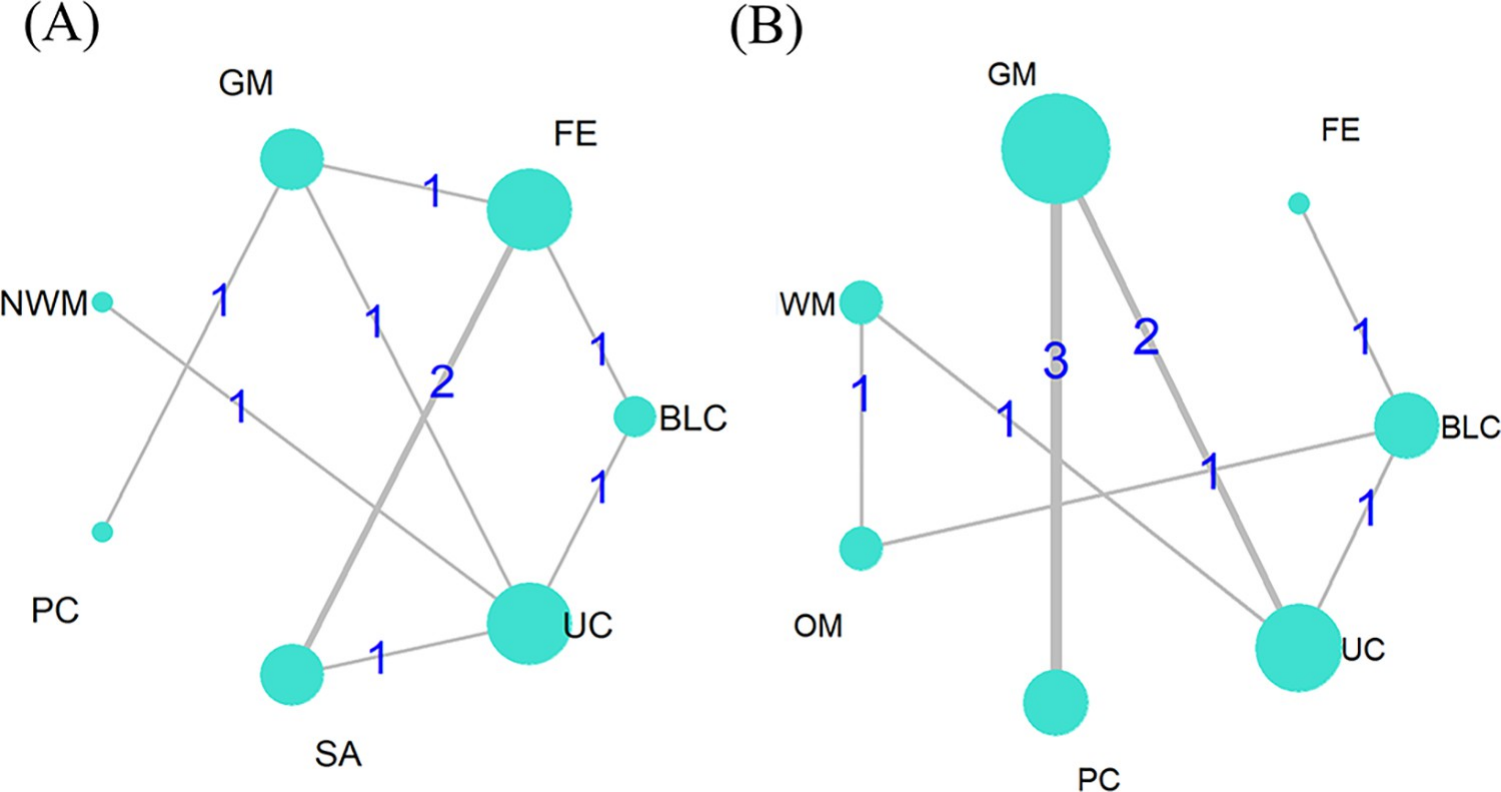
Network plot. (**A**) Clinical effectiveness rate, (**B**) Circumference of the elbow joint. Note: SA = simple acupuncture; BLC = blood-letting and cupping; GM = gentle moxibustion; NWM = needle-warming moxibustion; FE = functional exercises; OM = oral medicine; PC = pneumatic injection; UC = usual care.

NMA was only possible for the outcomes of clinical effectiveness rate and circumference of the elbow joint. However, the transitivity and consistency of the networks for the other outcomes could not be evaluated; hence, pairwise meta-analysis was the only approach employed. The network plots for each intervention that is a part of each NMA are shown in Fig 2. 9 trials [26–28, 32, 35, 37, 39, 40, 41] with 7 interventions (SA, BLC, GM, NWM, FE, PC, UC) were included in the network plot of the clinical effectiveness rate (Fig 2(A)). 10 studies [29–31, 34, 36–40, 43] containing 7 therapies (BLC, GM, NWM, FE, OM, PC, UC) were shown in the network plot of the elbow joint circumference (Fig 2(B)).

### Quality assessment

**Fig 3:**
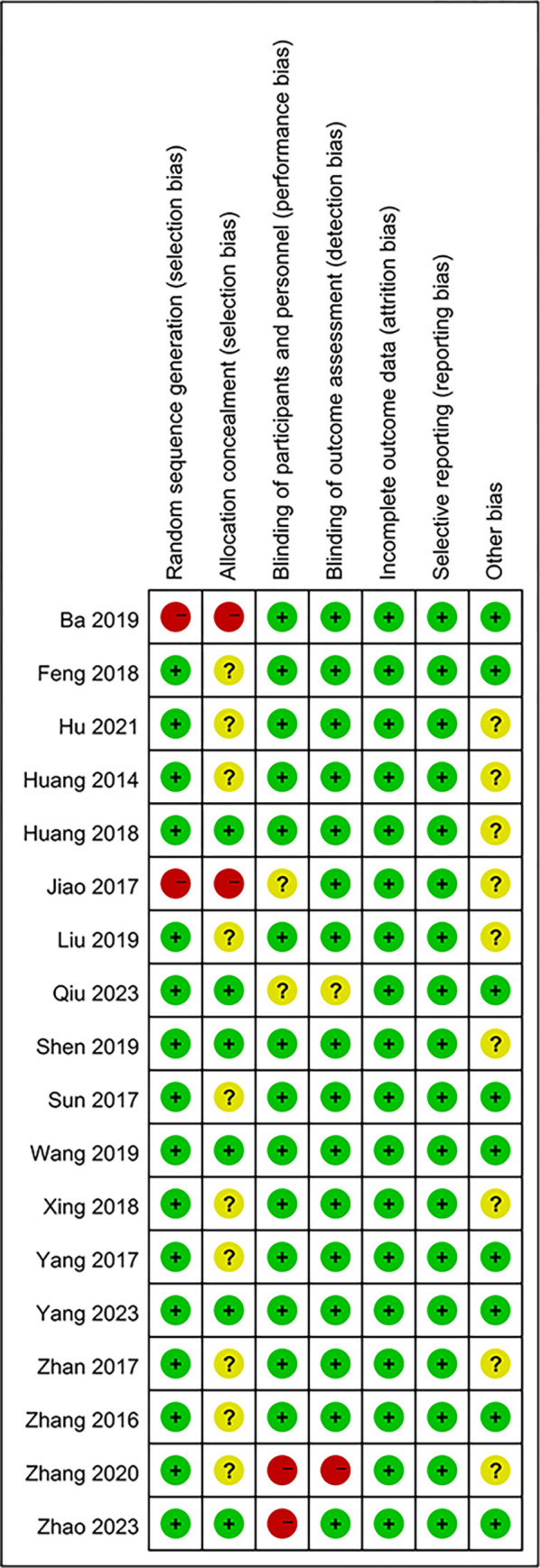
Risk assessment of bias.

Based on the collected literature, we evaluated the risk of bias. The result is given in Fig 3. 2 trials did not mention randomness. Due to unclear reporting, 6 trials reported the specific allocation concealment scheme, while the other studies did not. 2 trials were evaluated as having high risk because there was no report of any blind method, but the evaluator judged that it would not affect the measurement of objective outcomes. All the studies fully reported the predetermined outcomes. Most of them did not explain other bias risks and were rated as "unclear risk".

### Meta-analysis

#### Clinical effectiveness rate (pairwise meta-analysis and NMA)

According to the pairwise meta-analysis, GM (1 study, OR = 7.27, 95%CI: 1.49 to 35.46, P = 0.014), BLC (1 study, OR = 16.43, 95%CI: 3.21 to 84.00, P = 0.001) and SA (2 studies, OR = 6.78, 95%CI: 2.68 to 17.11, P = 0.000) were superior to FE in improving clinical effectiveness rate. However, there was no significant difference when comparing GM with PC (1 study, OR = 3.33, 95%CI: 0.52 to 21.28, P = 0.203). Compared with UC, the clinical effectiveness rate was significantly increased in the GM group (1 study, OR = 5.13, 95%CI: 1.27 to20.81, P = 0.022) and the BLC group (1 study, OR = 2.35, 95%CI: 1.01 to 5.46, P = 0.047). Additionally, it was not found that NWM (1 study, OR = 7.25, 95% CI: 0.82 to 64.46, P = 0.076) and SA (1 study, OR = 4.75, 95%CI: 0.48 to 46.91, P = 0.182) performed better than UC (S1 Fig).

**Fig 4:**
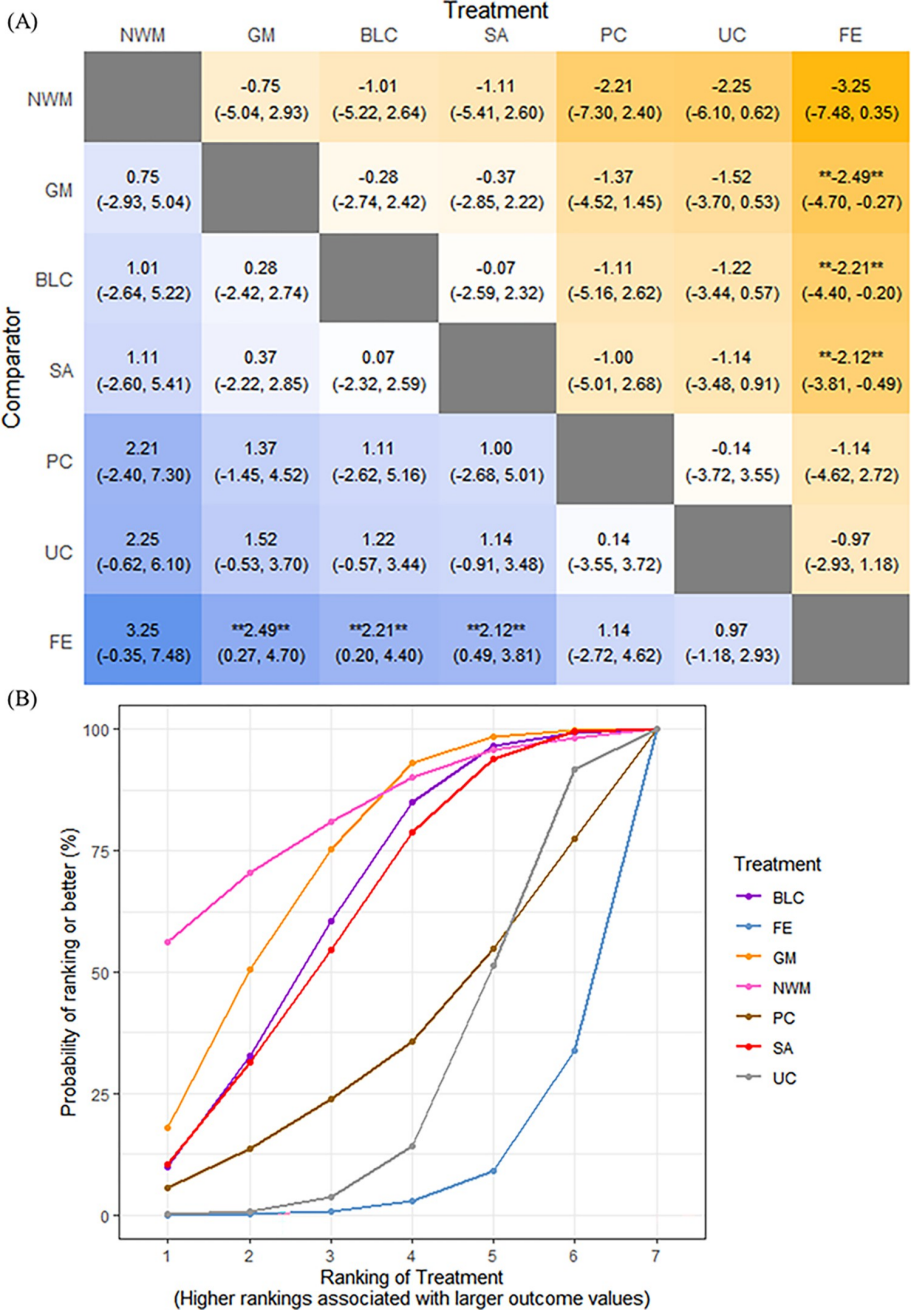
NMA results in the Clinical effective rate. (**A**) Heat plot. (**B**) SUCRA plot. Note: ** indicates a significant result.

The results of the NMA showed that GM, BLC, and SA significantly increased the clinical effectiveness rate compared with FE. In the meanwhile, GM was better than FE (Fig 4(A)). Based on the SUCRA of the clinical effectiveness rate, NWM (83.2%) was the optimal intervention, followed by GM (71.8%), BLC (64.7%), and SA (61.2%) (Fig 4(B)).

#### Average arm circumference (pairwise meta-analysis only)

6 studies [27, 29–31, 36, 39] with 291 participants explored variations in the average arm circumference. Pairwise meta-analysis showed that NWM (1 study; WMD = -1.00, 95%CI: -1.58 to -0.42, P = 0.001) was more efficacious than OM in reducing average arm circumference. However, there was no significant difference when comparing GM with PC (4 studies, WMD = -0.64, 95%CI: -1.30 to 0.03, P = 0.060, I^2^ = 0%). Additionally, it was not found that BLC (1 study, WMD = -0.74, 95% CI: -1.91 to 0.43, P = 0.215) performed better than FE (S2 Fig).

#### Circumference of the elbow joint (pairwise meta-analysis and NMA)

The pairwise meta-analysis revealed no differences between GM and PC (3 studies; WMD = -0.40, 95%CI: -1.27 to 0.47, P = 0.362, I^2^ = 0%), BLC and FE (1 study; WMD = -0.90, 95%CI: -2.33 to 0.53, P = 0.216). Compared with UC, NWM (1 study, WMD = -8.06, 95%CI: -11.56 to -4.56, P = 0.000) and BLC (1 study, WMD = -1.70, 95%CI: -2.51 to -0.89, P = 0.000) both showed significant advantages in decreasing the circumference of the elbow joint. Additionally, it was found that BLC (1 study, WMD = -1.49, 95% CI: -2.60 to -0.38, P = 0.009) performed better than OM. We used a random-effect model to compare GM with UC (P = 0.108). Due to limited research and difficulty in conducting sensitivity analysis, high heterogeneity (I^2^ = 88.2%) might differ significantly from the severity of lymphedema in the patients included in this study (S3 Fig).

**Fig 5:**
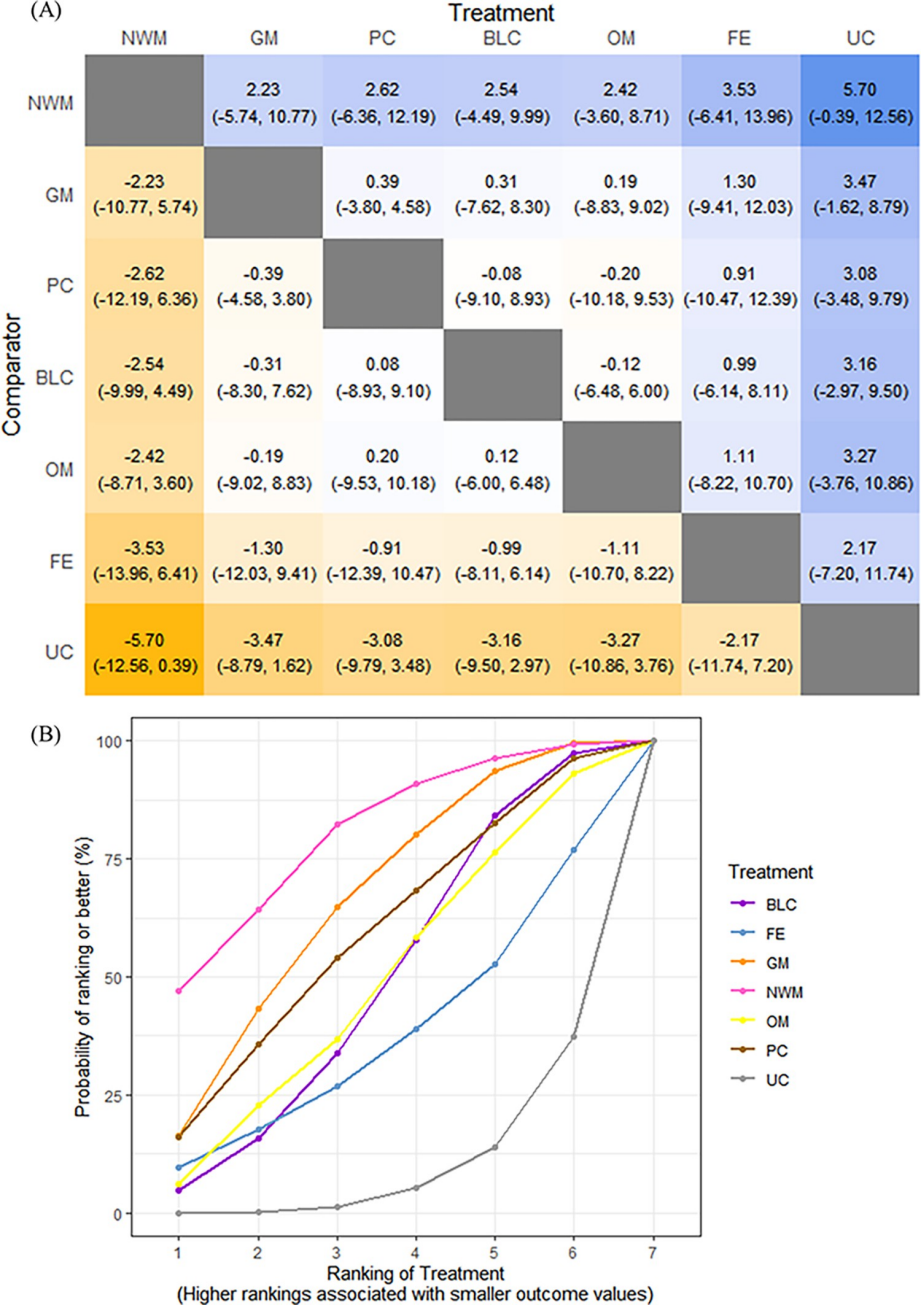
NMA results in the Circumference of the elbow joint. (**A**) Heat plot. (**B**) SUCRA plot. Note: ** indicates a significant result.

The NMA results indicated that no acupuncture and moxibustion therapies could reduce the circumference of the patient’s elbow (Fig 5(A)). Furthermore, according to the ranking of the SUCRA plot, the optimal intervention was NWM (82.2%), which was followed by GM (57.8%), and BLC (53.7%) (Fig 5(B)).

#### VAS score for swelling (pairwise meta-analysis only)

The VAS score for swelling was reported in 6 trials [27, 28, 30, 31, 36, 42]. As the pairwise meta-analysis results indicated, GM (4 studies, WMD = -1.19, 95%CI: -1.52 to -0.87, P = 0.000, I^2^ = 21.1%) was superior to PC in decreasing the swelling score. NWM performed greater than UC (1 study, WMD = -0.98, 95%CI: -1.73 to -0.23, P = 0.010), but not greater than PA (1 study, WMD = -0.73, 95%CI: -1.61 to 0.15, P = 0.105) (S4 Fig).

#### VAS score for pain (pairwise meta-analysis only)

3 studies [35, 37, 39] reported VAS scores for pain. Results of a pairwise meta-analysis revealed that when compared to UC, BLC had significant effects on reducing VAS pain score (1 study, WMD = -1.75, 95%CI: -2.24 to -1.26, P = 0.000). When comparing to FE, SA (1 study, WMD = -1.22, 95%CI: -1.71 to -0.73, P = 0.000) performed better in decreasing the VAS score of pain, while BLC (1 study, WMD = -0.42, 95%CI: -1.12 to 0.28, P = 0.242) did not (S5 Fig).

#### Adverse events

5 studies [29, 30, 33, 36, 42] reported that 10 patients had adverse events, 7 of which were related to acupuncture and moxibustion treatments. The specific adverse events included local skin discomfort (2 cases), mild scald caused by positional changes (2 cases), Flushing and itching (1 case), xerostomia (1 case), and swelling (1 case). No patients withdrew from the study due to adverse reactions. We found that the GM method had the highest probability of adverse events.

### Assessment of publication bias

In our study, the heterogeneities of most merged results were not high, indicating that our results had a certain degree of stability and reliability. The comparison-adjusted funnel plot of the circumference of the elbow joint lacked visual symmetry. However, Egger’s test showed no statistical difference in both outcomes (P = 0.130), indicating no significant publication bias in this outcome(S6 Fig). As for other outcomes, publication bias couldn’t be assessed through a funnel plot because their analysis included no more than 10 studies.

## Discussion

### Main findings

Patients with breast cancer are more likely to get postoperative lymphedema as a result of surgical techniques, local radiation, injury, and infection, which can lead to limb dysfunction and have a severe long-term impact on the patient’s quality of life. At present, Western medicine mainly uses comprehensive therapies (function exercise, compression, manual massage, and so on) to control swelling, but these methods have limited efficacy, and cost human and financial resources. Practical and effective treatments need to be explored. Acupuncture and moxibustion methods have been widely used in treating BCRL and had good efficacy. However, there might be differences in the efficacy of different acupuncture and moxibustion methods. Therefore, we carried out this network meta-analysis to investigate the efficacy and safety of various acupuncture and moxibustion methods in improving BCRL. Due to the use of different methodologies, scales, and measurement results in the RCTs included in this study, we only conducted both network meta-analysis and pairwise meta-analysis on 2 main outcomes (clinical effectiveness rate and circumference of the elbow joint). In contrast, only a pairwise meta-analysis was conducted on the other outcomes. In terms of the clinical effectiveness rate, the network meta-analysis results were consistent with the pairwise meta-analysis results. GM, BLC, and SA had significant improvements in the clinical effectiveness rate when compared to FE. In reducing the average arm circumference, the pairwise meta-analysis showed that only NWM was superior to OM. In the network meta-analysis of the circumference of the elbow joint, no significant differences were found in the efficacy of different treatments. However, pairwise meta-analysis results indicated that NWM and BLC were both better than UC. and GM has a significant effect in reducing the circumference of the elbow joint. The pairwise meta-analysis provided similar results. In addition, NWM and BLC acupuncture and moxibustion were better than UC in VAS score for swelling and pain, but other acupuncture and moxibustion methods had no obvious effect.

NWM demonstrated the highest likelihood of being ranked as the best method in terms of the clinical effectiveness rate and the circumference of the elbow joint, despite having a relatively small sample size. This is partially consistent with the conclusions of a recently published network meta-analysis [44]. NWM is a method that combines SA and GM to treat medical conditions [45]. SA has been widely used to treat cancer, arthritis, and other diseases [46, 47]. Some researchers claimed that acupuncture stimulation had the effect of relieving pain and regulating anti-inflammatory indicators (reducing pro-inflammatory cytokines as well as increasing anti-inflammatory cytokines) [48, 49]. Meanwhile, GM is second only to NWM in improving clinical effectiveness. The thermal effect of GM can dilate local capillaries, enhancing local lymphatic circulation and distribution [50], and by providing warm stimulation suitable for the human body, patients maintain a calm emotional, and healthy psychological state [51]. Multiple studies have shown that NWM relieves symptoms by introducing the thermal stimulation of moxibustion through acupuncture into the deep tissues of the affected limbs [52, 53]. The combination of acupuncture and moxibustion may produce a better therapeutic effect than the single use [54]. Infrared thermography suggests that the higher the body temperature in the deep layer of the affected limb, the more likely the improvement of lymphedema (lymph mainly flows to the deep layer) will be [55]. This may be one of the reasons why NWM is more effective than SA or GM. Previous studies have also proposed that NWM can probably promote lymphangiogenesis by upregulating the expression level of vascular endothelial growth factor C (VEGF-C), thereby facilitating the absorption of local tissue edema and inflammation [26, 56].

BLC refers to the method of using a sterile needle to quickly puncture the skin in the area, followed by cupping to release the appropriate amount of blood [57]. Pairwise meta-analysis suggested that BLC was superior to usual measures in all outcomes except VAS scores of swelling. This is inconsistent with previous study [58]. Perhaps due to the limited use of BLC treatment for BCRL in clinical practice, and insufficient sample size to support this [59]. Notably, EA is a treatment in which a small amount of electricity is applied to the needle after acupuncture is applied to prevent and treat diseases. However, Due to differences in measurement methods and the current situation of combining traditional Chinese medicine decoctions in clinical practice, only one trial using EA was included [60]. So, the specific efficacy of EA cannot be determined. EA may be used to stimulate the vagus nerve by pulsing radio waves to regulate immune function to promote recovery [49]. However, we found that in our searches, EA was mainly used in combination with decoctions of traditional Chinese medicine, so the direct effect of EA could not be ascertained.

The mechanism of point combination was relatively complex. In our study, the selection principle appeared to be a combination of local and distal points. Quchi (LI.11) is the most frequently used acupuncture point in the local (upper limb), followed by Ashi points. Quchi (LI.11) is at the bend and depression of the elbow joint, which is a large joint that is easily accessible to the upper limbs in daily life [61]. Pro-inflammatory gene expression can be reduced by stimulation and associated nerve development can be accelerated and controlled. Ashi points can be considered sensitive in the pathological condition’s hypersensitive state, which is located in the most painful part of the skin [62]. Zusanli (ST.36) is the most commonly used distal acupuncture point. It is an important health point, thanks to its powerful regulatory effect on various systems in the human body, including nerves, the immune system, and the endocrine system [29]. Based on the "holistic" concept, the combination of local and distal points can not only directly stimulate the affected area, but also further treat it by regulating the whole body, which is also a major feature of Traditional Chinese Medicine [63].

Regarding adverse events related to acupuncture and/or moxibustion therapies, we found that local skin discomfort is the most common one. In addition, patients who use the GM method may experience more adverse events, including local skin discomfort, mild scald caused by positional changes, Flushing and itching, xerostomia, and swelling. This may be related to the different feelings of each patient towards thermal stimulation, as well as the impact of moxa smoke [64]. However, adverse effects are not significant in their intensity. Overall, the side effects of acupuncture and/or moxibustion treatments are mild and acceptable to patients.

A follow-up survey of 3 studies [30, 34, 42] from 1 month to 4 months showed that edema recurred in some patients shortly after acupuncture and/or moxibustion treatment, but all had fewer cases of recurrence compared to control measures (OM and PC). The lack of long-term follow-up studies makes it hard to conclude substantial implications from acupuncture and/or moxibustion therapies in treating BCRL and which acupuncture and/or moxibustion therapy has the best long-term effect.

### Strengths and limitations

In our understanding, our research seems to be the latest to explore the most effective and safe acupuncture and/or moxibustion therapy for treating BCRL, which may be beneficial for making reasonable treatment decisions for BCRL because of a shortage of gold-standard treatments and significant medical burden. Secondly, we named the acupuncture and moxibustion methods in this study according to the international standard terminologies on traditional Chinese medicine [65] issued by the World Health Organization in 2022. Besides, to limit the impact of other measures on the results, we excluded acupuncture and/or moxibustion therapies used in combination with Chinese herbal medicine.

However, it is important to consider the following limitations. Firstly, the number of databases searched was Limited, and we only included 18 trials with 9 treatments. On this account, the majority of comparisons depended on 1–2 studies, which may not be detrimental to the reliability of the primary outcomes. Secondly, the included trials did not perform well in terms of methodological quality, and more than half of the studies had relatively small sample sizes. Therefore, it is possible that the reported effect sizes were exaggerated in these trials. Thirdly, the outcomes have not been standardized either, which has significantly impacted our data consolidation and analysis. This may be due to the lack of standardized treatments for BCRL. Finally, the small number of included studies makes it challenging for us to organize subgroup analysis.

### Future perspectives

In our meta-analysis, NMW is currently the best method, but in addition to the selection of methods, the acupoint selection and stimulation are also crucial. And, the selection of acupoints varies depending on the patient’s situation, so future research on specific acupoint combinations and mechanisms is needed. In addition, it is difficult to do a pooled analysis since different studies have employed the circumference of various sections of the affected limb as the outcome. It is advised that the measuring location and calculation method be standardized for future research. BCRL typically has a longer chronic progression, and in addition to the short-term improvement in clinical results, more evidence is needed to confirm whether acupuncture and/or moxibustion therapies have long-term effects. We encourage future researchers to conduct RCTs with high-quality, large sample sizes, and multi-center to validate the available evidence.

## Conclusion

Based on our results, acupuncture and moxibustion interventions have great potential to improve the BCRL of patients with breast cancer. Particularly NWM has significant advantages in improving the clinical effectiveness rate of BCRL patients, reducing the arm circumference of affected limbs, and reducing the sense of swelling and pain. NWM combines the advantages of acupuncture and moxibustion, which are simple, economical, safe, and effective. By stimulating fixed acupoints while applying a warming effect to the deep layers of the skin, it can improve the patient’s lymphatic circulation, relieve edema, alleviate pain, and achieve better clinical efficacy.

## Supporting information

S1 Fig The pairwise meta-analysis of clinical effectiveness rate. (PDF)

S2 Fig The pairwise meta-analysis of average arm circumference. (PDF)

S3 Fig The pairwise meta-analysis of the circumference of the elbow joint. (PDF)

S4 Fig The pairwise meta-analysis of VAS swelling score. (PDF)

S5 Fig The pairwise meta-analysis of VAS swelling score. (PDF)

S6 Fig The comparison-adjusted funnel plot. (PDF)

S1 File Related data for this study. (XLSX)

S1 Table The PRISMA network meta-analysis checklist. (PDF)

S2 Table Comprehensive search strategies. (PDF)

S3 Table The full name of acupoints abbreviations. (PDF)
