# Supplementary material for: Comparison efficacy and safety of acupuncture and moxibustion therapies in breast cancer-related lymphedema: A systematic review and network meta-analysis
Source: PLoS One. 2024 May 14;19(5):e0303513. doi: 10.1371/journal.pone.0303513 (PMC11093363; doi:10.1371/journal.pone.0303513)

S3 Fig. The pairwise meta-analysis of clinical effectiveness rate.

(1) GM vs FE

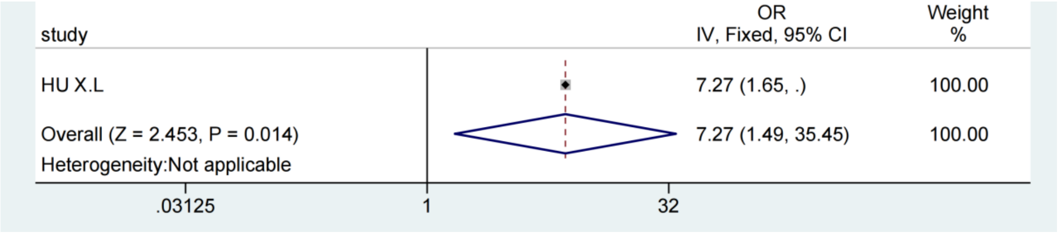

(2)GM vs UC

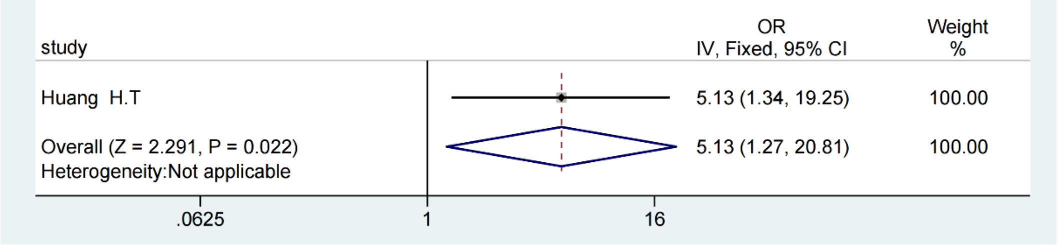

(3) GM vs PC

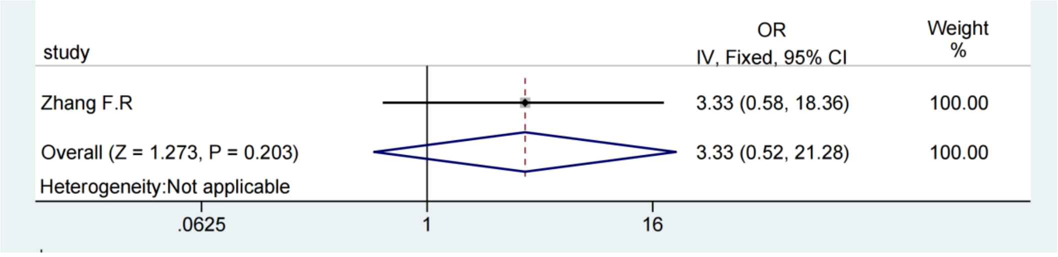

(4) BLC vs FE

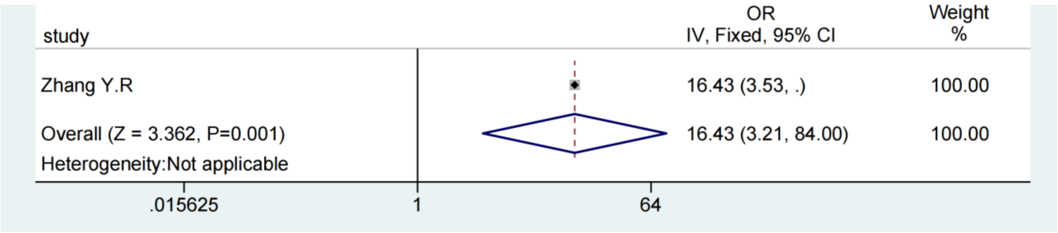

(5) BLC vs UC

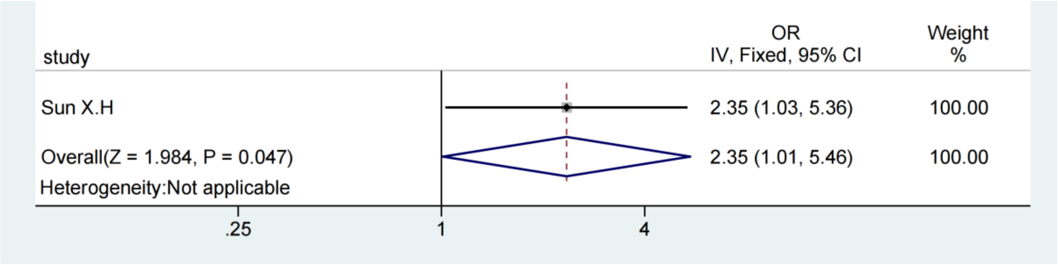

(6) NWM vs UC

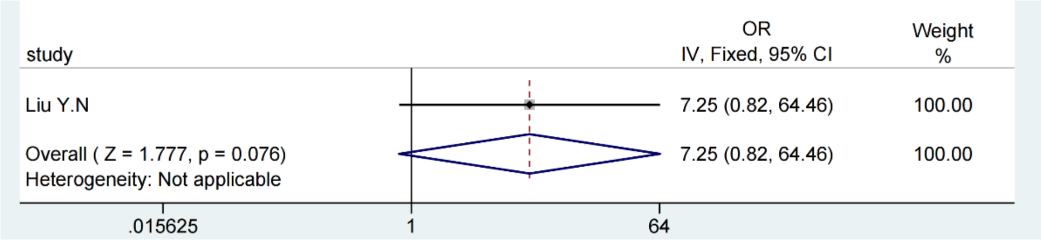

(7) SA vs UC

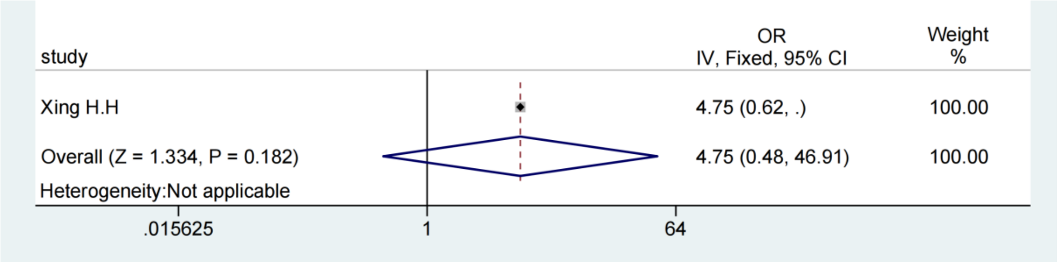

(8) SA vs FE

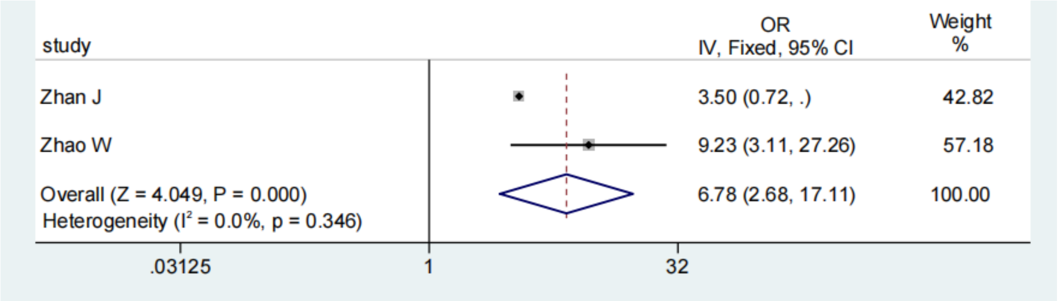

Supplement: S1 Fig — (PDF) [file pone.0303513.s001.pdf]
