# Supplementary figures and images for: Comparison efficacy and safety of acupuncture and moxibustion therapies in breast cancer-related lymphedema: A systematic review and network meta-analysis
Source: PLoS One. 2024 May 14;19(5):e0303513. doi: 10.1371/journal.pone.0303513 (PMC11093363; doi:10.1371/journal.pone.0303513)

## S4 Fig. The pairwise meta-analysis of average arm circumference.

### (1) NWM vs OM

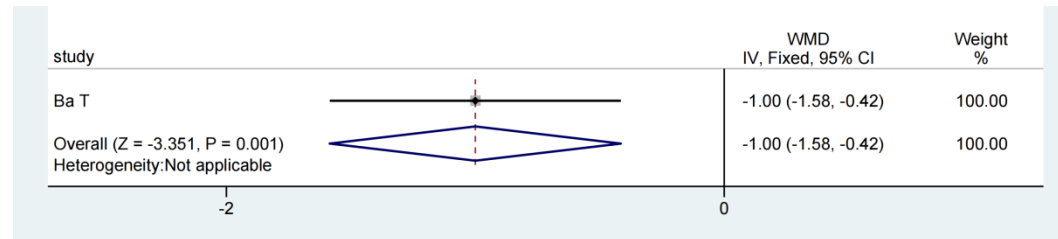

### (2) BLC vs FE

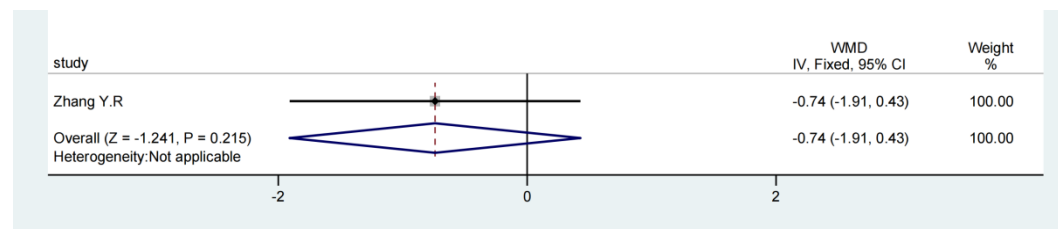

### (3) GM vs PC

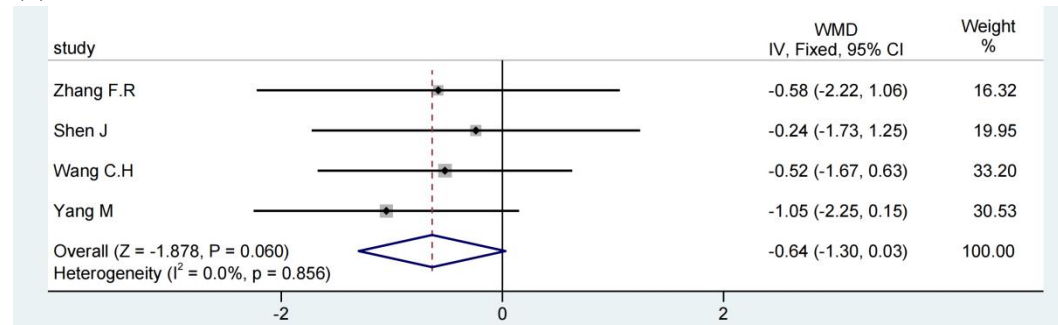

Supplement: S2 Fig — (PDF) [file pone.0303513.s002.pdf]

**S6 Fig. The pairwise meta-analysis of VAS swelling score.**

**(1) GM vs PC**

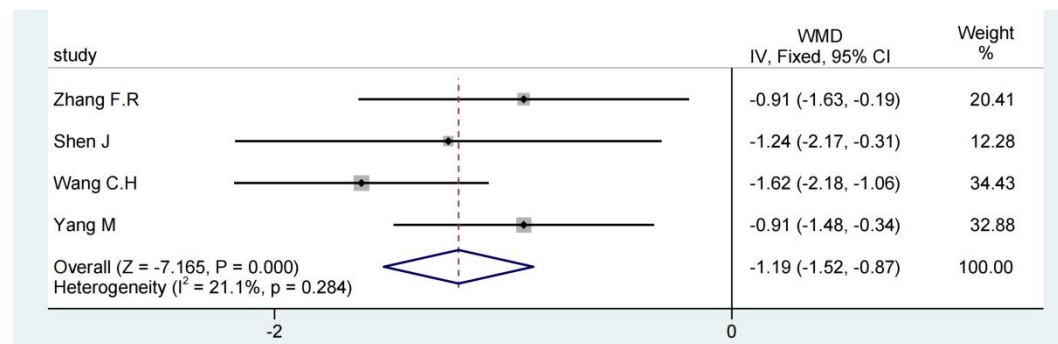

**(2) NWM vs UC**

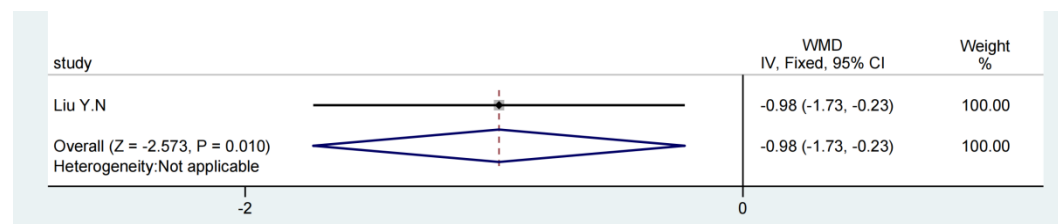

**(3) NWM vs PA**

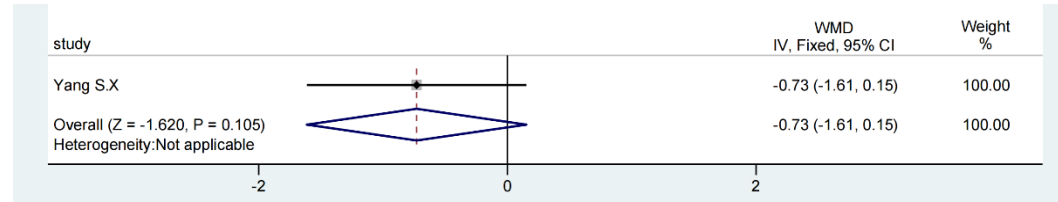

Supplement: S4 Fig — (PDF) [file pone.0303513.s004.pdf]

**S7 Fig. The pairwise meta-analysis of VAS pain score**

**(1) BLC vs UC**

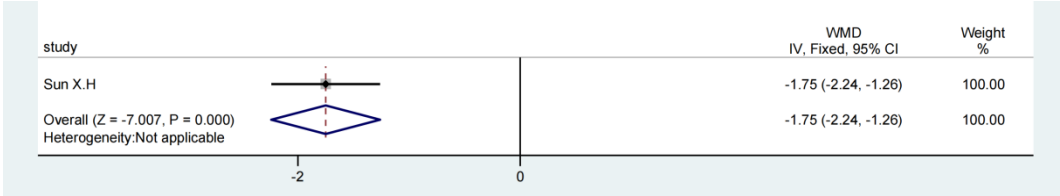

**(2) BLC vs FE**

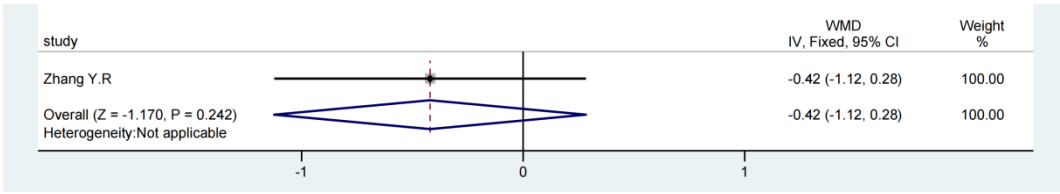

**(3) SA vs FE**

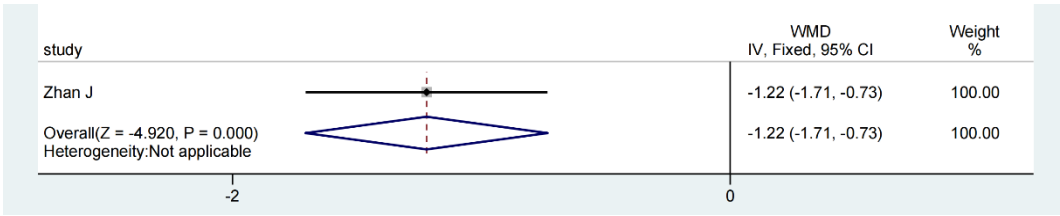

Supplement: S5 Fig — (PDF) [file pone.0303513.s005.pdf]

**S8 Fig.** The comparison-adjusted funnel plot.  
**(1) the circumference of the elbow joint**

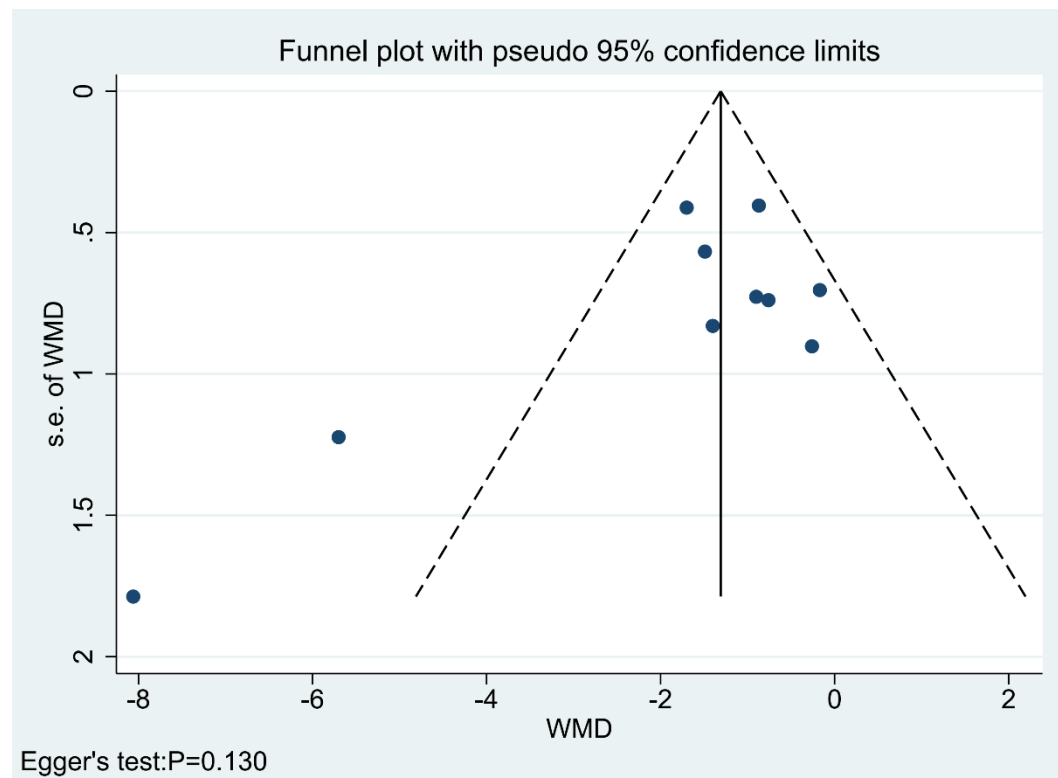

Supplement: S6 Fig — (PDF) [file pone.0303513.s006.pdf]
