# Supplementary material for: Comparison efficacy and safety of acupuncture and moxibustion therapies in breast cancer-related lymphedema: A systematic review and network meta-analysis
Source: PLoS One. 2024 May 14;19(5):e0303513. doi: 10.1371/journal.pone.0303513 (PMC11093363; doi:10.1371/journal.pone.0303513)
Supplement: S3 Fig — (PDF) [file pone.0303513.s003.pdf]

S5 Fig. The pairwise meta-analysis of the circumference of the elbow joint.

(1) GM vs PC

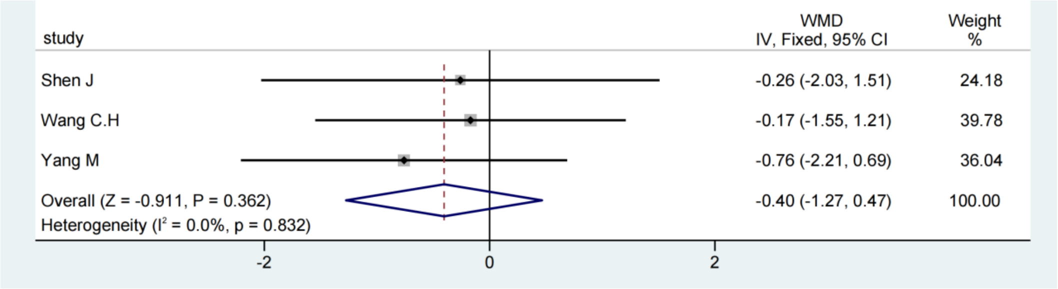

(2) GM vs UC

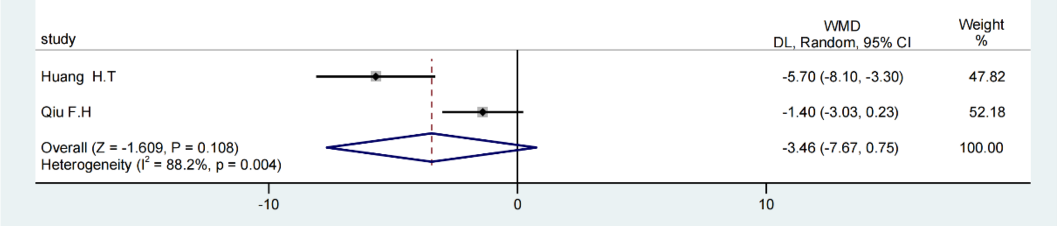

(3) BLC vs FE

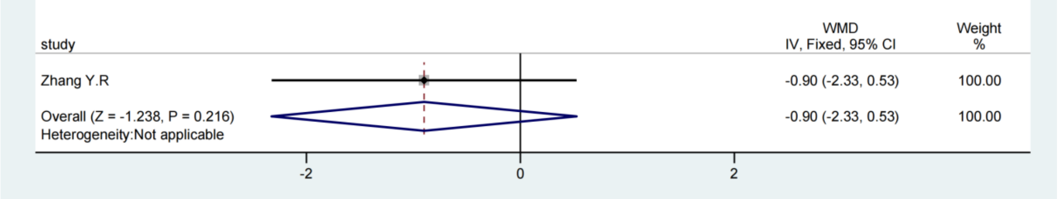

(4) BLC vs UC

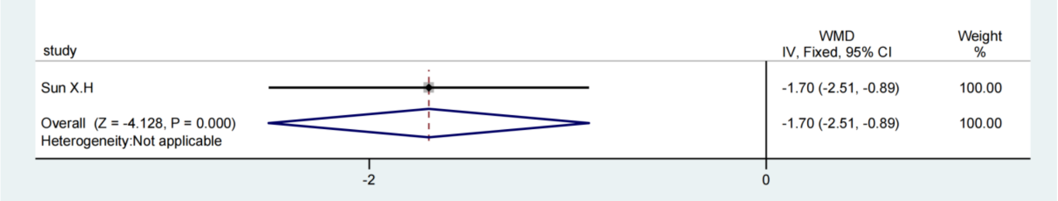

(5) BLC vs OM

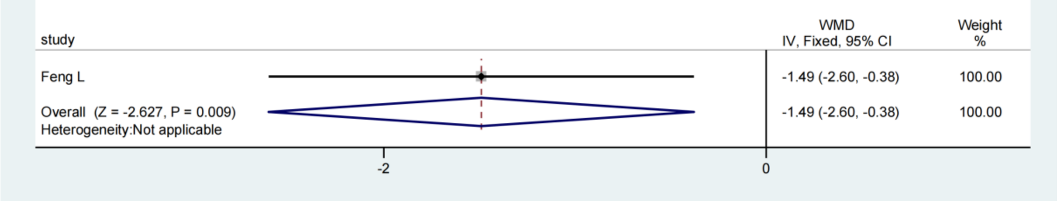

(6) NWM vs UC

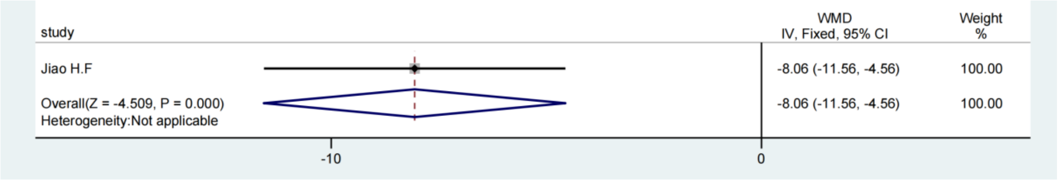

(7) NWM vs OM

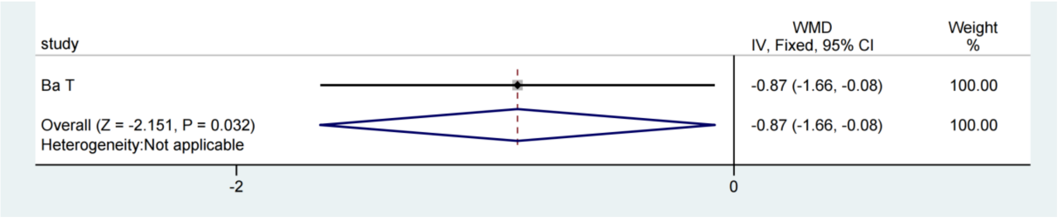
study
WMD
IV, Fixed, 95% CI
Weight
%
Ba T
-0.87 (-1.66, -0.08)
100.00
Overall (Z = -2.151, P = 0.032)
Heterogeneity:Not applicable
-0.87 (-1.66, -0.08)
100.00
-2
0
