# Supplementary material for: Comparison efficacy and safety of acupuncture and moxibustion therapies in breast cancer-related lymphedema: A systematic review and network meta-analysis
Source: PLoS One. 2024 May 14;19(5):e0303513. doi: 10.1371/journal.pone.0303513 (PMC11093363; doi:10.1371/journal.pone.0303513)
Supplement: S2 Table — (PDF) [file pone.0303513.s009.pdf]

S2 Table. Comprehensive search strategies.

PubMed

Number	Search terms	Results
#1	"breast neoplasms "[Mesh]	
#2	“breast neoplasms”[Title/Abstract] OR “breast tumors”[Title/Abstract] OR “breast cancer”[Title/Abstract]	
#3	#1 OR #2	
#4	“lymphoedema”[Title/Abstract]OR “Lymphedem*”[Title/Abstract] OR “lymph* edem*” [Title/Abstract] OR “lymph* oedem*” [Title/Abstract] OR “lymph* swell*” [Title/Abstract] OR “elephantias*” [Title/Abstract] OR “BCRL” [Title/Abstract]	
#5	(#1 OR #2) AND #3	
#6	“acupuncture”[Mesh]	
#7	"moxibustion"[Mesh]	
#8	“acupuncture” [Title/Abstract] OR “acupunctur*” [Title/Abstract] OR “acupress*”[Title/Abstract] OR“needling”[Title/Abstract] OR “electric-acupuncture”[Title/Abstract] OR “Zhenjiu”[Title/Abstract]OR “meridian*”[Title/Abstract] OR “Ching Lo”[Title/Abstract] OR “jing luo”[Title/Abstract] OR “moxibustion*”[Title/Abstract] OR “needl*”[Title/Abstract]	
#9	#6 OR #7 OR #8	
#10	#5 AND #9 Publication date: inception up to December 1st, 2023	82

Web of Science

Number	Search terms	Results
#1	TOPIC: “breast neoplasms” OR “breast tumors” OR “breast cancer”	
#2	TOPIC: “lymphoedema” OR “Lymphedem*” OR “lymph* edem*” OR “lymph* oedem*” OR “lymph* swell*” OR “elephantias*” OR “BCRL*”	
#3	#1 AND #2	
#4	TOPIC: “acupuncture” OR “moxibustion” OR “acupunctur*” OR “acupress*” OR “needling” OR “electric- acupuncture” OR “Zhenjiu” OR “meridian*” OR “Ching Lo” OR “jing luo” OR “moxibustion*” OR “needl*”	
#5	#3 AND #4 Publication date: inception up to December 12th, 2022	123

Embase

Number	Search terms	Results
#1	‘breast neoplasms’:ti,ab,kw or ‘breast tumors’:ti,ab,kw or ‘breast cancer’:ti,ab,kw	
#2	‘lymphoedema’:ti,ab,kw OR ‘Lymphedem*’:ti,ab,kw OR ‘elephantias*’:ti,ab,kw OR ‘BCRL’:ti,ab,kw	
#3	#1 AND #2	
#4	‘acupuncture’:ti,ab,kw OR ‘moxibustion’:ti,ab,kw OR ‘acupress*’:ti,ab,kw OR ‘needling’:ti,ab,kw OR ‘electric-acupuncture’:ti,ab,kw OR ‘Zhenjiu’:ti,ab,kw OR ‘meridian*’:ti,ab,kw OR ‘Ching Lo’:ti,ab,kw OR ‘jing luo’:ti,ab,kw	

#5	#3 AND #4 Publication date: inception up to December 12th, 2022	72

CNKI

Number	Search terms	Results
#1	(SU= ‘breast neoplasms’ OR SU=‘breast tumors’ OR SU=‘breast cancer’) AND (SU=‘lymphoedema’ OR SU=‘elephantias*’) AND (SU=‘acupuncture’ OR SU=‘moxibustion’ OR SU=‘acupress*’ OR SU=‘needling’)<br>Publication date: inception up to December 12th, 2022	116

Wangfang data

Number	Search terms	Results
#1	(SU= ‘breast neoplasms’ OR SU=‘breast tumors’ OR SU=‘breast cancer’) AND (SU=‘lymphoedema’ OR SU=‘elephantias*’) AND (SU=‘acupuncture’ OR SU=‘moxibustion’ OR SU=‘acupress*’ OR SU=‘needling’)<br>Publication date: inception up to December 12th, 2022	245

VIP

Number	Search terms	Results
#1	(ti,kw= ‘breast neoplasms’ OR ti,kw=‘breast tumors’ OR ti,kw=‘breast cancer’) AND (ti,kw=‘lymphoedema’ OR ti,kw=‘elephantias*’) AND (ti,kw=‘acupuncture’ OR ti,kw=‘moxibustion’ OR ti,kw=‘acupress*’ OR ti,kw=‘needling’)<br>Publication date: inception up to December 12th, 2022	67

SinoMed

Number	Search terms	Results
#1	(ti,kw= ‘breast neoplasms’ OR ti,kw=‘breast tumors’ OR ti,kw=‘breast cancer’) AND (ti,kw=‘lymphoedema’ OR ti,kw=‘elephantias*’) AND (ti,kw=‘acupuncture’ OR ti,kw=‘moxibustion’ OR ti,kw=‘acupress*’ OR ti,kw=‘needling’)<br>Publication date: inception up to December 12th, 2022	181
