# Supplementary material for: Comparison efficacy and safety of acupuncture and moxibustion therapies in breast cancer-related lymphedema: A systematic review and network meta-analysis
Source: PLoS One. 2024 May 14;19(5):e0303513. doi: 10.1371/journal.pone.0303513 (PMC11093363; doi:10.1371/journal.pone.0303513)
Supplement: S3 Table — (PDF) [file pone.0303513.s010.pdf]

S10. The full name of acupoints abbreviations

abbreviations	The full name
LI	The large intestine meridian of hand-Yangming
SP	The spleen meridian of foot-Taiyin
ST	The stomach meridian of foot-Yangming
CV	Conception Vessel
GB	Gallbladder meridian of foot shaoyang
HT	Heart meridian of hand shaoyin
LR	Liver meridian of foot queyin
TE	Triple energizer meridian of hand-Shaoyang
SI	The small intestine meridian of hand-Taiyang
LU	Lung meridian of hand taiyin
BL	The bladder meridian of foot-Taiyang
PC	The pericardium meridian of hand-Jueyin

In order of appearance.
